# Evaluation of deep sedation effects and respiratory aspects of remimazolam besylate in elderly patients undergoing fiberoptic bronchoscopy

**DOI:** 10.3389/fmed.2025.1543866

**Published:** 2025-03-28

**Authors:** Dongmei Ma, Li Li, Fuyi Han, Jianhong Xu, Cheng Zhang

**Affiliations:** ^1^Department of Anesthesiology, the Fourth Affiliated Hospital of School of Medicine, and International School of Medicine, International Institutes of Medicine, Zhejiang University, Yiwu, Zhejiang, China; ^2^Department of Urology, the Fourth Affiliated Hospital of School of Medicine, and International School of Medicine, International Institutes of Medicine, Zhejiang University, Yiwu, Zhejiang, China

**Keywords:** remimazolam besylate, fiberoptic bronchoscopy, elderly, deep sedation, hypoxemia

## Abstract

**Background:**

The geriatric population, especially individuals over 65 years old with comorbidities classified by the ASA (American Society of Anesthesiologists) grading system, requires careful sedation management during flexible bronchoscopy (FB) to reduce the heightened risks of complications. Hypoxemia is a particularly critical concern in this demographic, leading to considerable morbidity, mortality, and increased healthcare costs. This study focuses on comparing the incidence of sedation-related hypoxemia and other adverse events between remimazolam besylate and propofol during FB procedures, aiming to enhance patient safety and optimize sedation practices in this vulnerable population.

**Methods:**

This prospective observational cohort study compared the incidence of hypoxemia and sedation-related adverse events between remimazolam besylate and propofol in 69 elderly patients (ASA I-III). Rigorous inclusion/exclusion criteria, clinical monitoring, and alongside comprehensive monitoring of clinical parameters and statistical analyses to ensure the validity of the results.

**Results:**

Hypoxemia occurred in 44.90% overall, with significantly lower incidence in remimazolam besylate cohort (29.42% vs. 60.00%; OR = 2.10, 95% CI 1.18–3.74, *p* = 0.017). Recovery to full alertness was prolonged with remimazolam (median 15[12.5–20] vs. 8[5.5–10] min; *p* < 0.001). A trend toward reduced hypotension was observed (17.65% vs. 37.14%, *p* = 0.0699), with no other significant safety differences.

**Conclusion:**

Remimazolam besylate demonstrates superior safety for elderly FB sedation, significantly reducing hypoxemia risk and accelerating recovery. These findings support its preferential use in geriatric sedation protocols, warranting further investigation to optimize clinical implementation strategies.

## Introduction

The geriatric population undergoing invasive procedures faces heightened sedation risks due to age-related physiological decline, particularly in cardiopulmonary compensatory mechanisms. This vulnerability is exacerbated during flexible bronchoscopy (FB), where sedation-induced respiratory depression frequently precipitates hypoxemia-a critical complication associated with increased morbidity and healthcare costs (1–5).

Current sedation protocols utilizing propofol demonstrate suboptimal safety profiles in elderly patients, with documented risks of hypotension (15–30% incidence) and oxygen desaturation events (6, 7). Emerging evidence from gastrointestinal endoscopy trials suggests remimazolam besylate, a novel ultra-short-acting benzodiazepine, may offer superior hemodynamic stability and reduced hypoxemia rates (8.7% vs. 24.1% vs. propofol) (8–12). However, critical knowledge gaps persist regarding its comparative efficacy in FB procedures specifically targeting elderly populations.

This prospective observational cohort study systematically evaluates hypoxemia incidence between remimazolam besylate and propofol in ASA I-III patients aged ≥65 undergoing FB. Through rigorous patient selection and multidimensional safety monitoring, we aim to establish evidence-based recommendations for geriatric sedation optimization.

## Materials and methods

This prospective observational cohort study was approved by the Ethics Committee of the Fourth Affiliated Hospital of Zhejiang University Medical College (YiWu, People’s Republic of China) (No: K2023055). The trial was registered with the Clinical Trial Registry in the 05/05/2023(No: ChiCTR2300071137). The period of enrolment was from May 2023 to March 2024. All patients gave written informed consent before enrolment.

**Figure 1:**
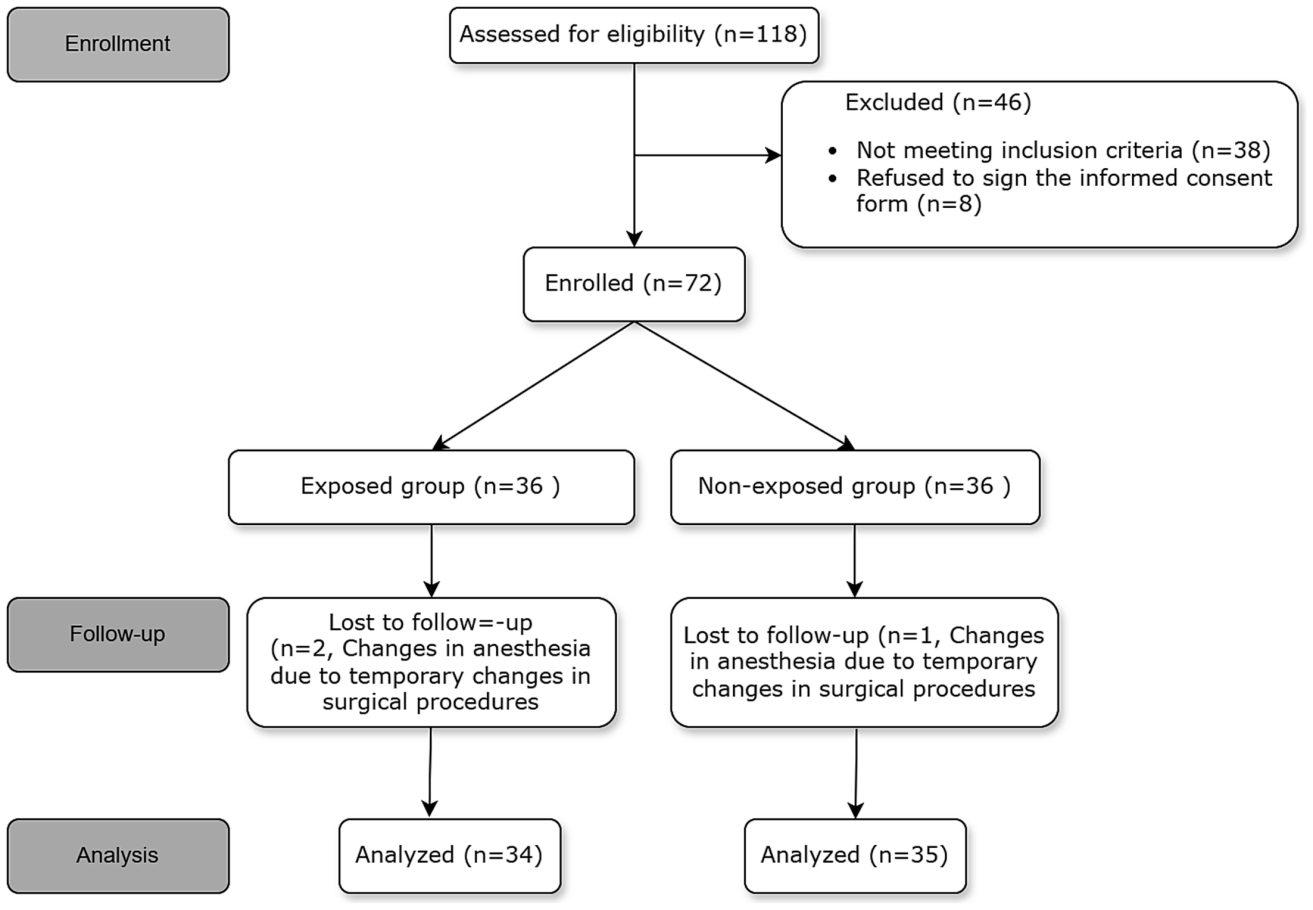
The flowchart showing the number of patients at each phase of the study.

The study recruited elderly participants aged 65 years and above who were undergoing FB and were classified under American Society of Anesthesiologists (ASA) grades I–III. The exclusion criteria encompassed the following: (1) ASA IV or Class V; (2) Body Mass Index (BMI) exceeding 35; (3) severe chronic obstructive pulmonary disease (COPD); (4) respiratory failure; (5) oxygen saturation (SPO2) below 90% when breathing room air; (6) lung function capacity of less than 15 mL/kg, with forced expiratory volume in 1 s (FEV1) less than 1,000 mL or FEV1/forced vital capacity (FVC) ratio below 35%; (7) Mallampati score greater than 4; and (8) known allergies to the study medication. Comprehensive information regarding the inclusion and exclusion criteria can be obtained from Figure 1.

Following the acquisition of informed consent, initial data collection was conducted for all participants, which included measurements of weight, vital signs, and Modified Observer Assessment of Alertness/Sedation (MOAA/S) scores. Prior to the procedure, an intravenous catheter of either 18 or 20-gauge was placed in the upper extremity. Participants enrolled in the study received a 2% lidocaine solution (10 mL) via atomization 30 min before sedation, with all endoscopic procedures conducted at the endoscopy center. Each patient underwent 5 min of preoxygenation using 100% oxygen at a flow rate of 6 L/min delivered through a facemask prior to the procedure. Monitoring protocols for sedated patients adhered to the American Society of Anaesthesiologists (ASA) guidelines, which included measuring blood pressure, heart rate (HR), pulse oxygen saturation, and end-tidal carbon dioxide levels using a Cardio cap device (Mindray). The exposed group received remimazolam besylate and non-exposed group received propofol. Analgesia was provided with 0.5 μg/kg fentanyl following the infusion of 500 mL of a 0.9% lactated Ringer’s solution. Subsequently, either 0.15 mg/kg of remimazolam besylate or 1 mg/kg of propofol was given according to group assignment. A skilled endoscopist performed bronchoscopy when the Modified Observer’s Assessment of Alertness/Sedation (MOAA/S) score dropped to ≤1. Lidocaine (100 mg of a 2% solution) was applied to the main airway during bronchoscopy. Sedation was sustained at a MOAA/S score of ≤1 throughout the procedure through the continuous infusion of 1 mg/kg/h of remimazolam besylate in the exposed group or propofol (4–6 mg/kg/h) in the non-exposed group. Supplementary doses of 2.5 mg of remimazolam besylate or 0.5 mg/kg of propofol were administered to address instances of coughing or body movement. The administration of medications was concluded upon completing the procedure, and oxygen supplementation was maintained at a flow rate of 6 L/min until the patient showed full alertness, which was confirmed by achieving a MOAA/S score of 5 in three consecutive evaluations.

### Respiratory safety monitoring

In instances of prolonged hypoxemia (SPO2 < 90%) lasting over 10 s, interventions such as elevating the mandibular angle and applying gentle thoracic compression were implemented. If hypoxemia persisted (SPO2 < 80% without spontaneous respiration), the bronchoscope was retracted, and manual ventilation was initiated until SPO2 levels increased above 95%. To ensure hemodynamic stability, vasoactive agents like phenylephrine, ephedrine, atropine, urapidil, and esmolol were used, maintaining parameters within a 20% deviation from baseline. After the bronchoscope was removed, patients were transferred to the Post-Anesthesia Care Unit (PACU) for recovery, where their MOAA/S scores were evaluated at five-minute intervals until full alertness was achieved. No pharmacological reversal agents (e.g., flumazenil) were administered. Remimazolam besylate’s rapid esterase-mediated metabolism ensures prompt recovery, and predefined rescue measures (e.g., manual ventilation) were prioritized to manage adverse events per protocol.

Throughout the study period, adverse events such as hypoxemia, hypotension, and bradycardia were closely monitored according to established criteria. Specifically, hypoxemia (13) was characterized by an SPO2 level falling below 90% for more than 30 s, while severe hypoxemia was defined as an SPO2 level dipping below 80% at any point.

The primary aim of the study was to investigate the frequency of hypoxemia, defined as SPO2 < 90% for more than 30 s, while secondary objectives included assessing severe hypoxemia (14) (Spo2 < 80%) and duration of full alertness (15) (MOAA/S score 5 for three consecutive occurrences).

### Statistical analysis

All analyses were performed using R 4.2.2 and MSTATA^1^ with two-tailed *α* = 0.05. Continuous variables were assessed for normality via the Kolmogorov–Smirnov test, reported as mean ± SD (normal distribution) or median [IQR] (non-normal). Group comparisons utilized Student’s *t*-test (parametric) or Mann–Whitney *U* test (non-parametric), while categorical variables were analyzed with Pearson’s *χ*^2^ test, multivariate logistic regression analysis for identifying independent factors associated with hypoxemia, and the significance level (*α* = 0.05).

## Results

A total of 118 patients underwent initial assessment, of whom 46 were subsequently excluded from the study. Among the excluded patients, 38 did not meet the inclusion criteria. This included 10 cases of severe chronic obstructive pulmonary disease (COPD), 5 cases with a body mass index (BMI) greater than 35, 8 cases with a peripheral capillary oxygen saturation (SpO₂) below 90%, 9 cases with a forced expiratory volume in 1 s (FEV1) below 1,000 mL, and 6 cases with a Mallampati score of grade 3–4. Additionally, 8 patients declined to provide informed consent.” Ultimately, 72 patients were included in the study, but 3 were later excluded due to surgical requirements necessitating a modification in anesthesia approach. This led to a final analysis involving 69 patients. The participant flow diagram is presented in Figure 1.

The demographic and clinical characteristics of the patients are presented in Table 1, demonstrating that there were no significant differences in the general characteristics such as age, weight, height, BMI, ASA score, and types of surgery between the two groups (*p* > 0.05).

**Table 1:** Patient characteristics and clinical results (*n* = 76).

Characteristics	Exposed (*N* = 34)	Non-exposed (*N* = 35)	All (*N* = 69)	*p*-value
Sex, *n* (%)				0.83
Male	27 (79.41%)	26 (74.29%)	53 (76.81%)	
Female	7 (20.59%)	9 (25.71%)	16 (23.19%)	
Age, y	73.32 (5.15)	71.37 (5.76)	72.33 (5.51)	0.14
BMI, (kg/m^2^)	22.74 (3.50)	23.31 (2.79)	23.03 (3.15)	0.46
Smoking, *n* (%)	16 (47.06%)	20 (57.14%)	36 (52.17%)	0.55
Drink, *n* (%)	10 (29.41%)	11 (31.43%)	21 (30.43%)	>0.99
History of diabetes, *n* (%)	5 (14.71%)	7 (20.00%)	12 (17.39%)	0.79
History of hypertension, *n* (%)	12 (35.29%)	13 (37.14%)	25 (36.23%)	>0.99
ASA physical status				>0.99
II, *n* (%)	14 (41.18%)	14 (40.00%)	28 (40.58%)	
III, *n* (%)	20 (58.82%)	21 (60.00%)	41 (59.42%)	
Type of surgery, *n* (%)				0.88
BAL	18(52.94%)	16(45.71%)	34(49.28%)	
Except BAL	16(47.06%)	19 (54.29%)	35(50.72%)	
Procedure time, (min)	12.50[10.00–28.75]	15.00 [14.00–20.00]	15.00[10.00–20.00]	0.44

Data were presented as number (percentage) for categorical variables, mean (SD) or median (interquartile range) for continuous variables. ASD was used to evaluate the balance of baseline information between groups. ASA, American Society of Anesthesiologists; ASD, absolute standardized difference; BMI, body mass index; SD, standard deviation; BAL, indicates bronchoalveolar lavage; Except BAL include EBUS and TBB; EBUS, endobronchial ultrasound; TBB, transbronchial biopsy.

**Figure 2:**
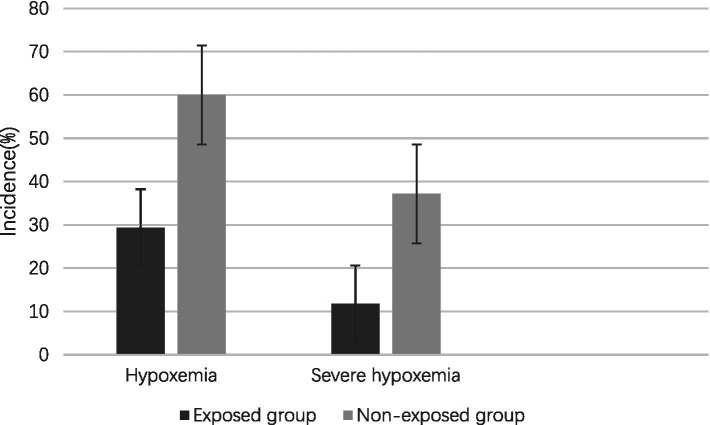
Incidence of hypoxemia and severe hypoxemia.

The study revealed an overall incidence of hypoxemia at 44.90%, with a lower prevalence observed in the exposed group compared to the non-exposed group (29.42% vs. 60.00%, OR 2.10, 95% CI 1.18–3.74, *p* = 0.017) prior to controlling for potential confounding variables. Similarly, the prevalence of severe hypoxemia was found to be 24.64%, with a lower occurrence in the exposed group compared to the non-exposed group (11.76% vs. 37.14%, OR 2.10, 95% CI 1.18–3.74, *p* = 0.017) before adjusting for confounding factors (Figure 2).

No statistically significant differences were observed in the occurrence of hypotension, bradycardia, or hypopnea between the two groups as indicated in Table 2. However, the incidence of hypotension was found to be 17.65%, with a lower occurrence observed in the exposed group compared to the non-exposed group (17.65% vs. 37.14%, *p* = 0.0699). Although the lack of statistical significance may be attributed to the small sample size, there is a trend suggesting potential clinical significance.

**Table 2:** Overall comparisons of adverse events after procedure.

Adverse events, *n* (%)	Exposed (*N* = 34)	Unexposed (*N* = 35)	*P* value overall
Hypotension	6(17.65%)	13(37.14%)	0.0699
Bradycardia	1(2.94%)	1(2.86%)	0.9834
Hypopnea	13(38.23%)	18(51.43%)	0.2707

Values were presented as number (percentage). A Pearson *χ*^2^ test or a Fisher exact was used to evaluate the associations between 2 groups.

**Figure 3:**
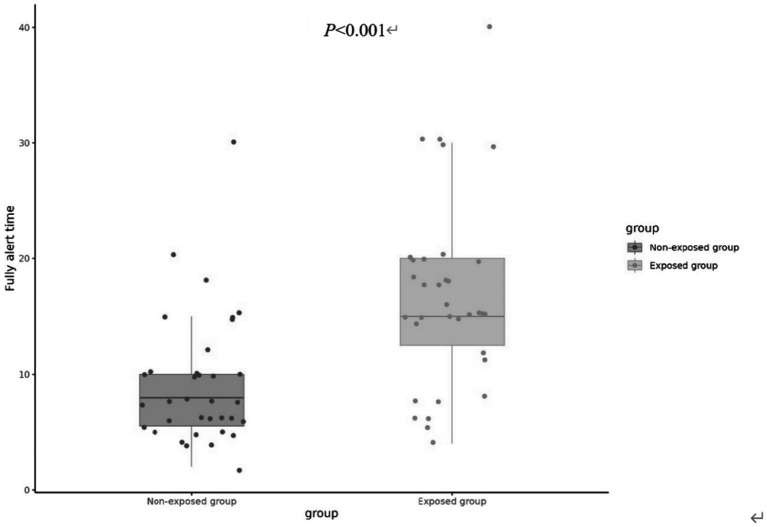
The fully alert time of two groups.

On the contrary, the fully alert time (MOAA/S score 5 for consecutive 3 times) was found to be significantly greater in the exposed group [15(12.5, 20) min] compared to the non-exposed group [8(5.5, 10) min] (*p* < 0.001), as illustrated in Figure 3.

In the analysis of perioperative factors and hypoxemia through multivariate logistic regression analysis, two significant and independent factors were identified as being correlated with the occurrence of hypoxemia (Table 3).

**Table 3:** Univariable logistic regression models for estimating the risk of hypoxemia.

Variables	*P*-value	OR (95%CI)
Group		
Exposed group	0.012*	0.28 (0.10 ~ 0.76)
Sex		
Female	0.642	1.30 (0.43 ~ 4.00)
BMI	0.233	1.10 (0.94 ~ 1.29)
Age, y	0.445	0.97 (0.89 ~ 1.06)
Procedure time	0.539	0.99 (0.94 ~ 1.03)
Type of surgery		
BAL	0.537	0.37 (0.14 ~ 0.98)
Smoking		
Yes	0.045*	0.37 (0.14 ~ 0.98)
Drink		
Yes	0.204	0.50 (0.17 ~ 1.46)
History of hypertension		
Yes	0.166	2.02 (0.75 ~ 5.47)
History of diabetes		
Yes	0.698	1.28 (0.37 ~ 4.45)
ASA		
III grade	0.775	1.15 (0.44 ~ 3.03)

BAL, indicates bronchoalveolar lavage; OR, odds ratio, CI, confidence interval. *p < 0.05, **p < 0.01, ***p < 0.001.

**Figure 4:**
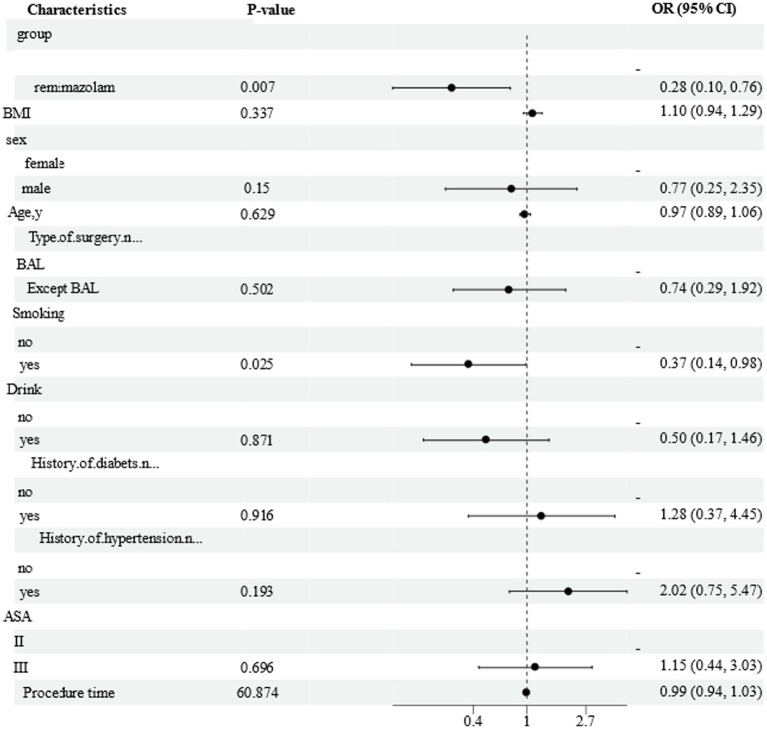
Forest plot of adjusted regression model for factors associated with hypoxemia. Figures show the reference value and OR (95%CI) for the levels of each variable; OR, odds radio.

In the multivariate analysis of these factors, remimazolam besylate exposure exhibited a lower risk of hypoxemia in comparison to propofol (OR 0.16, 95% CI 0.04–0.64, *p* = 0.010). Additionally, smoking was found to be associated with a reduced risk of hypoxemia when compared to non-smoking (OR 0.008, 95% CI 0.01–0.51, *p* = 0.008), as indicated in Figure 4.

## Discussion

Hypoxemia, characterized by abnormally low arterial oxygen levels (16), remains a critical risk for elderly patients undergoing sedation during flexible bronchoscopy (FB). This study revealed a significantly lower incidence of hypoxemia in the remimazolam besylate group compared to the propofol cohort (29.42% vs. 60.00%, *p* = 0.017), alongside prolonged recovery to full alertness (median 15[12.5–20] min vs. 8[5.5–10] min, *p* < 0.001). These findings align with prior research demonstrating remimazolam besylate’s favorable safety profile in procedural sedation (2, 17, 18), but extend its validation to elderly FB patients-a population historically underrepresented in such studies.

The observed reduction in hypoxemia incidence may stem from remimazolam besylate’s unique pharmacokinetics. Unlike propofol, which suppresses respiratory drive through GABA receptor potentiation (19), remimazolam besylate combines rapid esterase metabolism with selective α1-subunit binding (19, 20), potentially mitigating respiratory depression. This mechanism likely contributed to the lower rates of severe hypoxemia (11.76% vs. 37.14%, *p* = 0.017) and hypotension trends (17.65% vs. 37.14%, *p* = 0.0699) observed in our cohort.

These results resonate with earlier trials comparing remimazolam besylate to propofol in colonoscopy patients (5), where hypotension rates were halved (23.71% vs. 51.05%). However, our study uniquely highlights its efficacy in elderly patients with ASA I-III comorbidities-a demographic at heightened risk for sedation-related complications. The logistic regression further identified remimazolam besylate exposure as an independent protective factor against hypoxemia (OR 0.16, 95% CI 0.04–0.64, *p* = 0.010), reinforcing its clinical utility in this high-risk population.

The innovation of this study lies in its comparative analysis of remimazolam besylate and propofol regarding hypoxemia incidence during FB in elderly patients-a population often excluded from sedation trials (17, 18). While previous research focused on colonoscopy or general anesthesia (21, 22), our findings directly address the unmet need for optimized sedation protocols in geriatric bronchoscopy. This distinction is critical, as FB imposes unique respiratory challenges due to airway instrumentation and reduced functional reserve in elderly patients (2).

Notably, smoking emerged as a protective factor against hypoxemia (OR 0.008, 95% CI 0.01–0.51, *p* = 0.008), though this paradoxical finding requires cautious interpretation. The gender imbalance (76.81% male) and associated smoking prevalence (52.17%) may confound this association, necessitating further investigation into potential physiological mechanisms or selection biases.

Our findings align with growing evidence favoring deep sedation for complex endoscopic procedures (23), where reduced patient movement enhances procedural success. Despite concerns that deeper sedation increases hypoxemia risk (24), remimazolam besylate demonstrated superior safety, suggesting its pharmacokinetic advantages may offset traditional risk profiles.

Study limitations include the observational design and small sample size, which limit causal inferences and generalizability. Future multicenter RCTs with extended follow-ups are needed to validate these findings. Furthermore, further studies to explore the impacts of different sedative drugs on multiple aspects of the cardiovascular and nervous systems of elderly patients. Also, study how to optimize the drug combination and administration plan to better balance the sedation effect and reduce adverse reactions, especially for elderly patients with multiple comorbidities. Nevertheless, the consistency of our results with prior trials strengthens the argument for remimazolam besylate as a first-line sedative in elderly FB patients.

In summary, this study provides compelling evidence that remimazolam besylate is associated with a significantly reduced incidence of hypoxemia compared to propofol in elderly patients undergoing flexible bronchoscopy. These findings underscore the potential of remimazolam besylate as a safer sedation option, advocating for a re-evaluation of current sedation practices in this vulnerable population. The implications of this research extend beyond individual patient outcomes, suggesting that adopting remimazolam besylate could enhance overall clinical protocols, reduce healthcare burdens associated with sedation-related complications, and ultimately improve patient care in geriatric medicine.

## Conclusion

This study establishes, for the first time, that remimazolam besylate reduces hypoxemia incidence by over 50% compared to propofol in elderly patients undergoing FB-a high-risk population historically underrepresented in sedation research. By directly comparing these agents in a geriatric cohort with comorbidities, we demonstrate remimazolam’s dual advantages: superior respiratory safety (29.42% vs. 60.00% hypoxemia, *p* = 0.017) and hemodynamic stability, despite deeper sedation requirements. These findings redefine sedation paradigms for airway procedures in aging populations, addressing a critical gap in evidence-based protocols. Our results provide actionable insights for prioritizing its use in geriatric bronchoscopy to mitigate morbidity risks and optimize post-procedural recovery.

## Data Availability

The raw data supporting the conclusions of this article will be made available by the authors, without undue reservation.

## Footnotes

^1^ www.mstata.com

## References

[1] Zhang W Wang JL Fu S Zhou JM Zhu YJ Cai SN. Incidence of oxygen desaturation using a high-flow nasal cannula versus a facemask during flexible bronchoscopy in patients at risk of hypoxemia: a randomised controlled trial. BMC Pulm Med. (2022) 22:389. doi: 10.1186/s12890-022-02188-4, PMID: 36303179 PMC9615168

[2] Chhajed PN Glanville AR. Management of hypoxemia during flexible bronchoscopy. Clin Chest Med. (2003) 24:511–6. doi: 10.1016/s0272-5231(03)00050-9, PMID: 14535223

[3] Lu Z Zhou N Li Y Yang L Hao W. Up-down determination of the 90% effective dose (ED90) of remimazolam besylate for anesthesia induction. Ann Palliat Med. (2022) 11:568–73. doi: 10.21037/apm-22-89, PMID: 35249335

[4] Tan DD Gu J Li J Yu WQ Liu DX Zhao LJ. The effective doses of remimazolam besylate in the procedural sedation of endoscopic retrograde cholangiopancreatography. Ibrain. (2023) 9:290–7. doi: 10.1002/ibra.12072, PMID: 37786755 PMC10527792

[5] Chen S Wang J Xu X Huang Y Xue S Wu A. The efficacy and safety of remimazolam tosylate versus propofol in patients undergoing colonoscopy: a multicentered, randomized, positive-controlled, phase III clinical trial. Am J Transl Res. (2020) 12:4594–603. PMID: 32913533 PMC7476156

[6] Cao Y Chi P Zhou C Lv W Quan Z Xue FS. Remimazolam Tosilate sedation with adjuvant Sufentanil in Chinese patients with liver cirrhosis undergoing gastroscopy: a randomized controlled study. Med Sci Monit Int Med J Exp Clin Res. (2022) 28:e936580. doi: 10.12659/MSM.936580, PMID: 35706340 PMC9210946

[7] Chang Y Huang YT Chi KY Huang YT. Remimazolam versus propofol for procedural sedation: a meta-analysis of randomized controlled trials. PeerJ. (2023) 11:e15495. doi: 10.7717/peerj.15495, PMID: 37334113 PMC10269568

[8] Pastis NJ Yarmus LB Schippers F Ostroff R Chen A Akulian J. Safety and efficacy of Remimazolam compared with placebo and midazolam for moderate sedation during bronchoscopy. Chest. (2019) 155:137–46. doi: 10.1016/j.chest.2018.09.015, PMID: 30292760

[9] Rex DK Bhandari R Desta T MP DM Schaeffer C Etzkorn K. A phase III study evaluating the efficacy and safety of remimazolam (CNS 7056) compared with placebo and midazolam in patients undergoing colonoscopy. Gastrointest Endosc. (2018) 88:427–437.e6. doi: 10.1016/j.gie.2018.04.2351 29723512

[10] Chen SH Yuan TM Zhang J Bai H Tian M Pan CX. Remimazolam tosilate in upper gastrointestinal endoscopy: a multicenter, randomized, non-inferiority, phase III trial. J Gastroenterol Hepatol. (2021) 36:474–81. doi: 10.1111/jgh.15188, PMID: 32677707

[11] Doi M Morita K Takeda J Sakamoto A Yamakage M Suzuki T. Efficacy and safety of remimazolam versus propofol for general anesthesia: a multicenter, single-blind, randomized, parallel-group, phase IIb/III trial. J Anesth. (2020) 34:543–53. doi: 10.1007/s00540-020-02788-6, PMID: 32417976

[12] Xu C He L Ren J Zhou J Guo H Chen N. Efficacy and safety of remimazolam besylate combined with alfentanil in painless gastroscopy: a randomized, single-blind, parallel controlled study. Contrast Media Mol Imaging. (2022) 2022:7102293. doi: 10.1155/2022/7102293 36263002 PMC9553471

[13] Conway A Sutherland J. Depth of anaesthesia monitoring during procedural sedation and analgesia: a systematic review and meta-analysis. Int J Nurs Stud. (2016) 63:201–12. doi: 10.1016/j.ijnurstu.2016.05.004 27236824

[14] Frat JP Ricard JD Coudroy R Robert R Ragot S Thille AW. Preoxygenation with non-invasive ventilation versus high-flow nasal cannula oxygen therapy for intubation of patients with acute hypoxaemic respiratory failure in ICU: the prospective randomised controlled FLORALI-2 study protocol. BMJ Open. (2017) 7:e018611. doi: 10.1136/bmjopen-2017-018611, PMID: 29275345 PMC5770951

[15] Antonik LJ Goldwater DR Kilpatrick GJ Tilbrook GS Borkett KM. A placebo- and midazolam-controlled phase I single ascending-dose study evaluating the safety, pharmacokinetics, and pharmacodynamics of remimazolam (CNS 7056): part I. Safety, efficacy, and basic pharmacokinetics. Anesth Analg. (2012) 115:274–83. doi: 10.1213/ANE.0b013e31823f0c28, PMID: 22190555

[16] Henig NR Pierson DJ. Mechanisms of hypoxemia. Respir Care Clin N Am. (2000) 6:501–21. doi: 10.1016/s1078-5337(05)70087-3, PMID: 11172576

[17] Choe JW Chung MJ Park SW Oh D Han SY Yang MJ. Safety and efficacy of remimazolam versus propofol during EUS: a multicenter randomized controlled study. Gastrointest Endosc. (2024) 100:183–191.e1. doi: 10.1016/j.gie.2024.04.001, PMID: 38580132

[18] Ahmer W Imtiaz S Alam DM Ahmed K Sajid B Yousuf J. Remimazolam versus propofol for sedation in gastrointestinal endoscopy and colonoscopy within elderly patients: a meta-analysis of randomized controlled trials. Eur J Clin Pharmacol. (2024) 80:493–503. doi: 10.1007/s00228-024-03624-6, PMID: 38261005

[19] Masui K. Remimazolam: its clinical pharmacology and evolving role in anesthesia and sedation practice. Curr Opin Anaesthesiol. (2024) 37:344–51. doi: 10.1097/ACO.0000000000001384, PMID: 38841907

[20] Intzilaki CV Davodi J Vilmann P Møller AM. The clinical role of remimazolam: protocol for a scoping review. Acta Anaesthesiol Scand. (2024) 68:956–9. doi: 10.1111/aas.14421, PMID: 38561232

[21] Godoroja-Diarto D Constantin A Moldovan C Rusu E Sorbello M. Efficacy and safety of deep sedation and anaesthesia for complex endoscopic procedures-a narrative review. Diagn Basel Switz. (2022) 12:1523. doi: 10.3390/diagnostics12071523, PMID: 35885429 PMC9323178

[22] Lu K Wei S Ling W Wei Y Ran X Huang H. Remimazolam versus propofol for deep sedation/anaesthesia in upper gastrointestinal endoscopy in elderly patients: a multicenter, randomized controlled trial. J Clin Pharm Ther. (2022) 47:2230–6. doi: 10.1111/jcpt.13797, PMID: 36334013 PMC10100088

[23] Yong SH Lee SH Oh SI Keum JS Kim KN Park MS. Malignant thoracic lymph node classification with deep convolutional neural networks on real-time endobronchial ultrasound (EBUS) images. Transl Lung Cancer Res. (2022) 11:14–23. doi: 10.21037/tlcr-21-870 35242624 PMC8825650

[24] Hoshijima H Higuchi H Sato Boku A Shibuya M Morimoto Y Fujisawa T. Patient satisfaction with deep versus light/moderate sedation for non-surgical procedures: a systematic review and meta-analysis. Medicine. (2021) 100:e27176. doi: 10.1097/MD.0000000000027176, PMID: 34516514 PMC8428728

